# Poor Family Relationships in Adolescence and the Risk of Premature Death: Findings from the Stockholm Birth Cohort Study

**DOI:** 10.3390/ijerph16101690

**Published:** 2019-05-14

**Authors:** Susanne Alm, Sara Brolin Låftman, Hannes Bohman

**Affiliations:** 1 Swedish Institute for Social Research, Stockholm University, SE-10691 Stockholm, Sweden; 2 Department of Public Health Sciences, Centre for Health Equity Studies (CHESS), Stockholm University, SE-10691 Stockholm, Sweden; sara.brolin.laftman@su.se; 3 Department of Neuroscience, Child and Adolescent Psychiatry, Uppsala University, Box 593, SE-75124 Uppsala, Sweden; hannes.bohman@neuro.uu.se; 4 Department of Women’s and Children’s Health, Akademiska University Hospital, SE-75185 Uppsala, Sweden; 5 Stockholm Health Care Services, Stockholm County Council, 10422 Stockholm, Sweden

**Keywords:** adverse childhood experiences, family conflict, family discord, death, cohort, longitudinal, prospective

## Abstract

Poor family relationships during childhood have been shown to have long-term negative effects on an offspring’s health. However, few studies have followed the offspring to retirement age, and relatedly, knowledge about the link between poor family relationships and premature death is scarce. The aim of this study was to examine the association between poor family relationships in adolescence and the risk of premature death, even when considering other adverse childhood conditions. Prospective data from the Stockholm Birth Cohort study were used, with 2636 individuals born in 1953 who were followed up until age 65. Information on family relations was based on interviews with the participants’ mothers in 1968. Information on mortality was retrieved from administrative register data from 1969–2018. Cox proportional hazards regressions showed that poor family relationships in adolescence were associated with an increased risk of premature death, even when adjusting for childhood conditions in terms of household social class, household economic poverty, contact with the child services, parental alcohol abuse, and parental mental illness (Hazard Ratio (HR), 2.08, 95% Confidence Interval (CI), 1.40–3.09). The findings show that poor family relationships in adolescence can have severe and long-lasting health consequences, highlighting the importance of early interventions.

## 1. Introduction

Family conditions are central for children’s opportunities to lead a healthy life. One important aspect is the family social environment. According to attachment theory, a close relationship between the child and at least one caregiver is crucial for a positive emotional and social development [1]. A safe and supportive family environment during upbringing is likely to provide the child with emotional security and a sense of social integration which are conducive elements for a healthy development. Accordingly, family relationships affect child development but also the offspring’s mental and physical health across the life course [2]. One core aspect of family relationships is family cohesion, which Vandeleur et al. [3] have defined as “the degree of togetherness or closeness or emotional bonding that family members have toward one another” (p. 1205). Features of high family cohesion include warmth, affection, consistency, involvement, and connectedness [3,4]. Higher levels of family cohesion have been shown to be associated with higher well-being [3] and fewer mental health problems in adolescents [4,5], indicating that positive and strong familial bonds promote healthy development. Conversely, lower levels of family cohesion are an indicator of poor family relationships and may hinder a healthy development. Indeed, as theorized by Repetti et al. [2], families characterized by unsupportive and neglectful relationships and conflict constitute risky environments that may lead to worse mental and physical health across the life, operating through pathways such as stress, emotion processing, social competence, and risky health behaviors [2].

Prior studies have reported that various aspects of poor family relationships during childhood have long-term health consequences. Most of these earlier studies have focused on mental health outcomes. Weich et al. [6] showed, in a systematic review of 23 studies, that dysfunctional family relationships in childhood were associated with an increased risk of psychiatric disorders later in life. Similarly, in their analyses of two British cohorts, Morgan et al. [7] found that poor parent-child relationships predicted mental health problems in adulthood. Using data from a Finnish cohort study, Berg et al. [8] showed that problems in adolescent family relationships were associated with psychological distress in mid-adulthood. Poor family relationships may however also affect somatic health in adulthood. Using data from three British national birth cohort studies, Stewart-Brown et al. [9] demonstrated that poor parent-child relationships were associated with self-reported health problems or illnesses in adulthood. The measures of health problems were based on lists of common health problems and illnesses but the authors did not break down the analyses by specific health problems. Furthermore, Landstedt et al. [10] reported, using data from the Northern Swedish Cohort Study, that poor parental contact at age 16 was associated with an increased risk of reporting both internalizing symptoms and functional somatic symptoms up until the age of 42. However, few earlier studies of poor family relationships and later health outcomes have followed the offspring as long as to retirement age, and relatedly, knowledge about the effects of poor family relationships on premature death is scarce. Yet, given the obvious link between mental and somatic ill-health and mortality, it is reasonable to assume that poor family relationships may lead not only to adverse health but also to premature death.

The association between poor family relationships in childhood and adolescence and later adverse health outcomes may be understood through several mechanisms. One possible explanation involves stress. Being exposed to a dysfunctional family social environment can be presumed to constitute a chronic stressor [2,11,12,13], which may affect individuals’ mental and somatic health [14]. Another non-mutually exclusive possible pathway is through poor social relationships in adulthood. Dysfunctional family relationships may have a negative impact on the children’s emotional and social competence, which may in turn hinder a healthy development and ultimately affect health. For instance, children who experience adverse family relationships are more likely to develop behavior that may trigger conflict in interpersonal relationships [2]. Studies have shown that favorable family relationships in childhood were connected with better social relations in adulthood, in terms of more satisfaction with partner relationships, stronger family relations, and a lower risk of experiencing loneliness [15]. Conversely, dysfunctional family relationships in childhood were associated with poorer social relationships in adulthood [15,16]. There is a well-established link between supportive social relationships and better health [17] and even survival [18]. The association between poor family relationships and later health outcomes may also operate via a socioeconomic path [12]. For instance, studies have demonstrated a link between poor parental support and lower educational attainment [19] as well as with economic adversity in adulthood [12]. Socioeconomic status, in terms of educational attainment, social class, and income, is in turn clearly and inversely associated with ill-health and mortality [20,21,22].

Poor family relationships may co-occur with other adversities such as child abuse, parental alcohol abuse, and parental mental illness. Such adverse childhood experiences are linked with increased risks of later ill-health [13,23,24,25] and even death [13,26]. Poor family relationships also often co-occur with adolescent mental disorders [27,28,29], which constitute a well-known risk factor for both mental and somatic ill-health in adulthood [30,31,32,33]. Thus, when studying the association between poor family relationships in adolescence and health-related outcomes in adulthood, other adversities in the family as well as adolescent mental disorder should be taken into consideration.

The aim of the current study was to examine the association between poor family relationships in adolescence and the risk of premature death, even when taking into account other adverse childhood conditions that may be related with both poor family relationships and premature death. To this end, we used prospective data from the Stockholm Birth Cohort study, with 2636 individuals born in 1953 who were followed up through administrative registers until age 65.

## 2. Materials and Methods

### 2.1. Participants

The data were derived from the Stockholm Birth Cohort study (SBC). The SBC database has been created by combining two anonymized data sets [34,35]. The first comprises the Metropolitan Study database of all individuals (*n* = 15,117) born in 1953 and living in Stockholm 10 years later [36]. The Metropolitan database includes large amounts of both survey and register data. When the members of the Metropolitan were or would turn 13, they were asked to complete a questionnaire in school, asking about friendship relations, interest in school, and future plans. Two years later, when the cohort members were or would turn 15, interviews were also made with their parents, in a majority of cases their mothers, for a subsample of them (*n* = 3651). The questions to the mothers concerned their attitudes to education and their hopes for the future of their child, but also their views on child rearing and their estimations of the relations within the family. Out of 4021 sampled individuals for the Family Study, interviews were completed with 3651 (91%). Register data within the Metropolitan Study include information on social problems in the family of origin from the Social Authorities, but also information on health, income and occupation.

The second data set, to which the data from the Metropolitan study have been linked, is the so-called health, illness, income, and work database (HSIA), which comprises register data on all individuals living in Sweden in 1980 or 1990. The data include information on inter alia, incomes, social welfare recipiency, and health. With the combined database, it is possible to follow the individuals born in 1953 until they were 65 years of age (in 2018).

This study uses the information from the subsample of mothers interviewed in the Family Study within the Metropolitan study together with register information from the HSIA (*n* = 3476). The study sample is further restricted to those respondents who at the age of 15 years were living with both their biological parents and who had at least one sibling. This means that the study sample comprises 2636 individuals.

The Reproduction of Inequality through Linked Lives (RELINK) project, of which the current study is a part, has been ethically approved by the Regional Ethical Review Board of Stockholm (2017/34–31/5 and 2017/684–32).

### 2.2. Measures

#### 2.2.1. Dependent Variable

All-cause mortality: Annual information on (all-cause) mortality in adult age (16–65 years) was derived from the causes of death register administered by the Swedish National Board of Health and Welfare.

#### 2.2.2. Independent Variable

Family relations: The measure was constructed from four questions asked to the participant’s mother in the Family Study (when the respondents were 15 years old). The questions read:

1. ‘How would you describe the relationship between you and your son/daughter?’

2. ‘How would you describe the relationship between your husband and your son/daughter?’

3. ‘How would you describe the relationship between your son/daughter and his/her siblings?’

4. ‘How would you describe the relationship between you and your husband?’

For all questions there were five response categories: (1) ‘very good’, (2) ‘rather good’, (3) ‘neither good nor bad’, (4) ‘rather bad’, and (5) ‘very bad’. The scores on the four questions were reversed and summed to an index with the range 5–20, with higher values indicating better relations. The index showed good internal consistency (Cronbach’s alpha = 0.75). Due to the skewed distribution, with a majority of respondents reporting ‘very good’ or ‘rather good’ familial relations, the index was divided into three categories to indicate ‘good’ (scores 19–20), ‘intermediate’ (scores 16–18), and ‘poor’ (scores < 16) family relations. With this operationalization, participants at approximately the bottom decile of the index were classified as having poor family relations. The cutoff implied that this category had not necessarily reported ‘bad’ relations in an absolute sense. Yet, in a relative sense, their family relationships could be classified as poor.

#### 2.2.3. Controls

Gender: 0 = male, 1 = female.

Household social class: The indicator was based on the father’s, or, if information on this was missing, on the mother’s occupation, and coded into three categories: (1) upper non-manual, (2) lower non-manual, self-employed, and farmers, and (3) manual and unclassified. The data on household social class was collected in 1963, when the study participants were 10 years of age.

Household economic poverty: The measure was based on register information and indicates social welfare recipiency in the family of origin in the years 1953–1965, i.e., when the study participants were aged 0–12. A dummy was constructed, distinguishing those who did not receive any social welfare during the period (0), from those who received social welfare on at least one occasion during the period (1).

Contact with child services: If the family of origin had been in contact with the child services as a consequence of the behaviour of the study person at age 7–12 years, the study participant was coded 1, otherwise 0.

Parental alcohol abuse: If (at least one of) the study participant’s parents had been registered by the social authorities for alcohol abuse at any time point during the period 1953–1965, the participant was coded 1, otherwise 0.

Parental mental illness: If (at least one of) the study participant’s parents had been registered by the social authorities for mental illness at any time point during the period 1953–1965, the participant was coded 1, otherwise 0.

### 2.3. Data Analysis

First, we assessed the bivariate, descriptive associations between the categories of family relationships and the control variables and premature death. These are presented by percentages (%) and numbers (*n*) of death. In the multivariate analyses, Cox (proportional hazards) regression modelling was used, estimating the risk of mortality up to age 65 for individuals exposed to ‘good’, ‘intermediate’, and ‘poor’ family relations in adolescence. Hazard ratios (HR) with 95% confidence intervals (95% CI) are reported. Both unadjusted (bivariate) and fully adjusted models (adjusting for the full set of control variables) are presented.

## 3. Results

### 3.1. Main Results

Descriptive statistics of the study variables are provided in Table 1. Out of the 2636 individuals in the study sample, 234 (8.9 percent) died at ages 64–65 years (in February 2018). The mothers in the Family Study generally rated the family relationships in quite positive terms, but according to our categorization, 9.1 percent of the participants were subjected to poor family relations. As regards economic and social problems in the family of origin, 12.3 percent suffered from economic problems, whereas between 1.7–2.9 percent scored positive on the three indicators of social problems, i.e., contact with the child services as a consequence of the study individual’s behavior at age 7–12 years, parental alcohol abuse, and parental mental illness.

Results from Cox regressions predicting all-cause mortality are presented in Table 2. The first column gives the proportion and the number of deaths associated with each independent factor in the model. The second column presents the unadjusted estimates, whereas the third column gives the adjusted estimates for the full model.

First, regarding the unadjusted estimates, all factors included in the model were related to the risk of premature death, and all but parental mental illness were statistically significant at the 5% level. The strongest associations were found for those who had been in contact with the child services due to their behavior at age 7–12 and those who grew up with a parent abusing alcohol. As is well known, premature death was also considerably more common among men than women. Regarding family relations, compared to participants whose mothers stated ‘good’ such relations, both those whose mothers estimated them as ‘intermediate’ and even more so as ‘poor’ showed a significantly higher risk of premature death up to age 65.

When including all variables in the full model, naturally, some of the associations were attenuated, but interestingly, the strong results concerning family relationships remained rather unaffected. In other words, even when controlling for mental illness and alcohol abuse in the parental generation, as well as for economic difficulties in the family of origin and for early contact with the child services, those who experienced ‘poor’ (or even ‘intermediate’) family relations in adolescence were significantly more likely to have suffered from premature death, than those who enjoyed ‘good’ family relations. As for the controls, early contact with the child services and gender remained statistically significant also in the adjusted model.

### 3.2. Sensitivity Analyses

Subsequently, a number of sensitivity analyses were performed to test the robustness of the results. Firstly, the study’s main independent variable, i.e., the summary index of family relations, was broken down into its four original components: Relationship between (1) mother and child, (2) father and child, (3) siblings, and (4) mother and father. In these analyses, the categories ‘neither good nor bad’, ‘rather bad’, or ‘very bad’ on each question were coded as a ‘poor’ relation, ‘rather good’ was coded as ‘intermediate’, and ‘very good’ (which served as the reference category) was coded as a ‘good’ relation. The results from the analysis are displayed in Table A1 in Appendix A. As can be seen from the table, all four components of the summary index appear to have contributed to the effect of the family relations measure, albeit to somewhat differing degrees. The strongest association was found for the mother-child relationship, followed by the father-child relationship. Concerning the relationship between the parents, the association did not quite reach 5% significance, whereas concerning sibling relationships, only those whose mothers had estimated these to be ‘poor’ were significantly more likely to have suffered from premature death than those whose mothers had estimated them as ‘good’. In sum, however, as indicated by the good reliability of the summary index (Cronbach’s alpha = 0.75), all four types of family relations appear to have contributed to the association between family relations during adolescence and premature death up to age 65.

In a next step, the dependent variable was modified to test the robustness of the results. Although the number of cases was too limited to enable an analysis of cause-specific mortality, it did allow a categorization into ‘natural’ and ‘unnatural’ causes of death, respectively. Thus, out of the 234 cases of death, 133 were classified as ‘natural’ (ischemic heart disease, respiratory disease, cancer, infectious, and inflammatory disease etc.) and 71 were classified as ‘unnatural’ (accidents, alcohol- and drug-related death, and suicide). For the remaining 30 cases, a classification could not be made, either because information on cause of death was missing in our data (which, e.g., was the case for all cases from January 2017 and onwards) or because the code indicated that the cause of death could not be determined. The average age at death due to unnatural causes was 41 years; for natural causes, it was 53 years. Two Cox regression models were run, one estimating the risk of natural death, the other of unnatural death (see Table A2 and Table A3 in Appendix A). The results showed that family relations in adolescence were related both to natural and unnatural causes of death in adulthood. Regarding natural causes of death, only those whose mothers had reported poor family relations were significantly more likely to have died prematurely, compared to those whose mothers had reported good family relations. On the other hand, concerning unnatural deaths, both those with intermediate and those with poor family relations in adolescence were more likely to have died prematurely, compared to those enjoying good family relations in adolescence.

The aim of the third and final sensitivity analysis was to test the direction of the hypothesized relationship between poor family relations and premature death. This was done by adding an additional independent factor to the model. Theoretically, it could be the case that mental illness among the study participants in young age could cause both poor family relations in adolescence and premature death in adulthood. To test this, an indicator of in-patient psychiatric care in age 16–25 years was added to the model, while the dependent measure was restricted to deaths in age 26–65. The measure of in-patient psychiatric care was based on data from the hospital discharge register on individuals admitted for inpatient treatment with a psychiatric diagnosis at the age of 16–25 years. The variable was a dummy taking the value 1 if the individual had experienced at least one event of such treatment in the ages 16–25, otherwise 0. A total of 78 individuals (3%) of the study sample had such experiences. Restricting the dependent measure to deaths in age 26–65 reduced the number of deaths from 2636 to 2619. The results of the model are presented in Table A4 in Appendix A. The table shows that in-patient psychiatric care in adolescence and young adulthood was, unsurprisingly, positively associated with premature death in adulthood. However, as seen from the table, the introduction of the variable in the model only marginally affected the association between family relations and premature death. Thus, this analysis supports the assumption that a significant part of the association between poor family relations in adolescence and premature death in adulthood could be causal in the way predicted in the background section.

## 4. Discussion

Using unique, prospective data from the Stockholm Birth Cohort study, with individuals born in 1953 who were followed until the age of 65, the current study showed that poor family relationships in adolescence had an independent and long-lasting negative effect in terms of an increased risk of premature death. The associations were clear and robust even when adjusting for household social class in childhood and a range of adverse childhood experiences. Strikingly, in the adjusted analyses, only family relationships and contact with child services during childhood showed independent associations with premature mortality. The association between contact with child services and premature mortality is in line with previous studies of the same data material, which showed that out-of-home care in childhood was associated with an increased risk of premature mortality [37,38]. To the best of our knowledge, however, the link between poor family relations and premature death has not been shown in earlier research. Yet, our findings are in line with previous studies showing that poor family relations were associated with adult somatic and mental health problems [6,7,8,9,10]. Such health problems are, in turn, related to an increased risk of mortality.

Three different types of sensitivity analyses, modifying both the dependent and the main independent variable, as well as challenging the hypothesized direction of causality, indicated good robustness of the results. First, all the different studied family relations individually predicted premature death, albeit with somewhat varying strength. This indicates that suboptimal family relations in any form are related to poor outcome throughout the life course. The second set of sensitivity analyses showed that poor family relationships in adolescence were related both to unnatural and natural causes of death. This implies that poor family relationships seem to be associated with psychiatric disorder, which is a risk factor for mortality particularly due to unnatural, but also to natural, causes [39]. Finally, studies have shown a strong association between poor family relations and mental health problems such as depression in childhood [27,28]. Yet, our third sensitivity analysis showed that poor family relations predicted premature death when adjusting for psychiatric care in adolescence and early adulthood, indicating that poor family relations had an independent effect on adverse outcome in terms of premature death.

Family relationships are important for the well-being of the offspring. Attachment theory postulates that close relationships with at least one caregiver are crucial for child development [1]. High levels of family cohesion, in terms of strong familial bonds, warmth and connectedness, are associated with higher well-being and fewer mental health problems among adolescents [3,4,5]. Conversely, low levels of family cohesion are likely to be stressful and may accordingly have negative consequences for health. Indeed, it is possible to assume that poor family relationships constitute a chronic stressor, which causes an imbalance in the individual’s natural response to stress. According to McEwen’s theory about allostatic load, such imbalance affects the body through pathways related to the neuroendocrine, autonomic, immune, and metabolic systems [14], with adverse effects on both mental and somatic health that may ultimately lead to death. This theory might serve as one explanation for the somewhat astonishing finding that family relations in childhood were clearly associated with an increased risk of premature death. Other possible pathways between poor family relationships in childhood and premature death include poor social relationships and adverse socioeconomic conditions in adulthood. These conditions are associated with both mental and somatic disorders, that in turn are associated with mortality. It is a relevant task for future research to empirically investigate the mechanisms and pathways, in addition to disentangling the differing trajectories for natural and unnatural causes of death.

The main merit of this study is the prospective data material used, which has information on childhood conditions collected in the 1960s and yearly administrative register information on deaths up until 2018. The long follow-up time also enabled us to distinguish between unnatural and natural causes of death. It is also a strength that we were able to adjust for household social class in childhood and a range of adverse childhood conditions, i.e., household economic poverty, contact with child services, parental alcohol abuse, and parental mental illness. The finding that the association between poor family relationships in adolescence and premature death was robust even when adjusting for these potential confounders supports the assumption that poor family relationships in adolescence has an independent effect on adverse mortality. In addition, adjusting for psychiatric care in adolescence and early adulthood did not substantially affect the associations, indicating that adolescent mental disorder was not a confounder. It should however be emphasized that our measures of these other childhood adversities capture rather severe conditions. More specifically, our measures of household economic poverty, contact with child services, parental alcohol abuse, and parental mental illness were all based on information on formal contacts with the authorities, thus capturing the most severe end of these adversities. It is likely that adversities such as economic hardship and parental mental illness were present in several of the studied families, although not captured through our data. The same is true for psychiatric care in adolescence, which probably represents only a small portion of individuals with mental health problems, more specifically those with the most serious problems. Hence, while we can draw the conclusion that the association between poor family relationships and premature death was not explained by our measures of severe adversities, it cannot be ruled out that less severe adverse childhood conditions may be confounders in these associations. Accordingly, future studies of family relationships and mortality should include also measures of adverse childhood conditions based on survey information, which are likely to capture a wider array of adversities rather than only the severe end.

The study also has other limitations. The fact that it covered only individuals growing up in nuclear families with at least two children inhibits generalizations to other family types. Yet, we do not see any reasons why the association between poor family relationships and premature death would not also be found in individuals growing up in single-parent households or without siblings. Further, our measure of family relationships was not based on a previously validated scale. It included information from four rather generally phrased questions and the information was collected only at one point in time. The general nature of the questions implies that we were not able to assess specific aspects of poor family relationships, e.g., lack of warmth. Furthermore, the fact that the measure was based only on mothers’ reports may limit the validity. The fact that very few mothers reported to have poor relationships with their children is an indication of the subjective account of the measure. At the same time, it does not seem likely that reports based on parental information would be less valid than reports from the participants themselves. In any case, measures of family relationships based on responses from multiple informants would have been preferable. Furthermore, with regards to our family relationships measure, it should be kept in mind that our categorization captured ‘poor’ relationships in a relative rather than in an absolute sense. Future studies should assess the associations between explicitly adverse aspects of family relationships, e.g., family conflict, and premature mortality. Another limitation concerns our construct of household social class in childhood. The measure was based on an older form of socioeconomic classification, which was standard in Sweden up to the 1970s [40]. A more recent classification system would have allowed us to distinguish between a larger number of categories. However, when divided into three categories only, the measure is very similar to newer versions of socioeconomic categorizations. Finally, the fact that the study was based on a cohort born in Stockholm in 1953 limits the generalizability to populations in other societal and geographical contexts. Thus, further research based on data from other national contexts is recommended to corroborate the findings.

The findings that poor family relations have severe consequences throughout the lifespan have practical and clinical implications. The results that poor and intermediate family relations are associated with premature mortality suggest that evidence-based parenting programmes for the general population could be beneficial. In addition, since poor family relationships often co-occur with adolescent mental disorders [27,28] and possibly also with familial social adversities, family therapeutic interventions in specific groups could be valuable.

## 5. Conclusions

The current study showed that poor family relationships in adolescence can have severe and long-lasting consequences in terms of premature death. The associations between poor family relationships and premature death were strong and robust even when other adverse childhood conditions were taken into consideration. In the adjusted analyses, the two childhood conditions that showed independent associations with premature death were poor (and intermediate) family relationships and contact with child services. 

To the best of our knowledge, this study is among the first to investigate the association between poor family relationships in childhood and premature death. To corroborate these findings, further studies using validated measures of family relations and controlling for less severe adverse childhood conditions are needed. Future research should also scrutinize the mechanisms in these associations.

## Figures and Tables

**Table 1:** Descriptions of the study variables (*n* = 2636).

Variable	Mean	^a^
Family relationships (index)	17.9	1.9
	*N*	%
Deaths in adulthood (16–65 years)	234	8.9
Family relationships (categorical)		
Good	1268	48.1
Intermediate	1128	42.8
Poor	240	9.1
Gender		
Males	1355	51.4
Females	1281	48.6
Household social class		
Upper class/upper middle class	592	22.5
Intermediate/lower middle class/entrepreneur/farmer	1058	40.1
Working class/unclassified	986	37.4
Household economic poverty	325	12.3
Contact with child services	54	2.0
Parental alcohol abuse	45	1.7
Parental mental illness	76	2.9

^a^ Standard deviation.

**Table 2:** Associations between family relations in adolescence and all-cause mortality in adulthood. Hazard ratios (HR) and 95% confidence intervals (95% Confidence Intervals (CI)) from Cox (proportional hazards) regressions. Results statistically significant at the 5%-level are reported in bold. (*n* = 2636).

Variable	% Deaths (*n*)	Unadjusted		Adjusted	
		HR	95% CI	HR	95% CI
Family relations					
Good (ref.)	6.9 (87)	1.00	-	1.00	-
Intermediate	9.8 (111)	**1.45**	1.10–1.92	**1.43**	1.08–1.89
Poor	15.0 (36)	**2.27**	1.54–3.35	**2.08**	1.40–3.09
Gender					
Males (ref.)	11.1 (151)	1.00	-	1.00	-
Females	6.5 (83)	**0.57**	0.43–0.74	**0.56**	0.43–0.74
Household social class					
Upper non-manual (ref.)	5.9 (35)	1.00	-	1.00	-
Intermediate/lower non-manual/					
entrepreneur/farmer	8.5 (90)	1.46	0.99–2.16	1.34	0.91–1.99
Manual worker	11.1 (109)	**1.93**	1.32–2.82	1.50	1.00–2.24
Household economic poverty	15.1 (49)	**1.96**	1.43–2.69	1.47	1.00–2.14
Contact with child services	29.6 (16)	**4.21**	2.54–7.00	**2.59**	1.52–4.42
Parental alcohol abuse	22.2 (10)	**2.90**	1.54–5.47	1.84	0.93–3.66
Parental mental illness	14.5 (11)	1.71	0.93–3.13	0.85	0.44–1.65

## Appendix A

**Table A1:** Sensitivity analysis. Associations between family relations in adolescence and all-cause mortality in adulthood, breaking down the index of family relations into its four components: Mother-child relation, father-child relation, sibling relations and mother-father relation. Gender, SES, household economic poverty, contact with child services, parental alcohol abuse and parental mental illness controlled for in models reporting adjusted effects. Hazard ratios (HR) and 95% confidence intervals (95% CI) from Cox (proportional hazards) regressions. Results statistically significant at the 5%-level are reported in bold. (*n* = 2636).

Variable	% Deaths (*n*)	Unadjusted		Adjusted	
		HR	95% CI	HR	95% CI
Family relations					
Mother-child					
Good	7.3 (119)	1.00	-	1.00	-
Intermediate	11.1 (107)	**1.54**	1.19–2.00	**1.53**	1.18–1.99
Poor	18.6 (8)	**2.69**	1.31-5.50	**2.53**	1.21–5.29
Father-child					
Good	7.3 (110)	1.00	-	1.00	-
Intermediate	10.6 (109)	**1.47**	1.13-1.91	**1.44**	1.10–1.88
Poor	15.0 (15)	**2.15**	1.25-3.68	**1.84**	1.05–3.22
Sibling relations					
Good	7.6 (75)	1.00	-	1.00	-
Intermediate	8.8 (115)	1.17	0.87–1.56	1.13	0.84–1.51
Poor	12.5 (44)	**1.68**	1.16–2.44	**1.54**	1.06–2.24
Mother-father					
Good	7.1 (131)	1.00	-	1.00	-
Intermediate	10.7 (90)	**1.41**	1.07–1.84	1.28	0.98–1.68
Poor	14.0 (13)	**1.90**	1.07–3.35	1.73	0.98–3.08

**Table A2:** Sensitivity analysis. Associations between family relations in adolescence and mortality from natural causes of death. Hazard ratios (HR) and 95% confidence intervals (95% CI) from Cox (proportional hazards) regressions. Results statistically significant at the 5%-level are reported in bold. (*n* = 2636).

Variable	% Deaths (*n*)	Unadjusted		Adjusted	
		HR	95% CI	HR	95% CI
Family relations					
Good (ref.)	4.6 (58)	1.00	-	1.00	-
Intermediate	4.9 (55)	1.07	0.74–1.54	1.04	0.72–1.51
Poor	8.3 (20)	**1.86**	1.12–3.09	**1.71**	1.02–2.88
Gender					
Males (ref.)	5.8 (78)	1.00	-	1.00	-
Females	4.3 (55)	0.74	0.52–1.04	0.75	0.53–1.06
Household social class					
Upper non-manual (ref.)	3.7 (22)	1.00	-	1.00	-
Interm./lower non-manual/					
entrepreneur/farmer	5.2 (55)	1.41	0.86–2.31	1.31	0.80–2.16
Manual worker	5.7 (56)	1.55	0.94–2.53	1.27	0.75–2.13
Household economic poverty	7.7 (25)	**1.68**	1.09–2.60	1.57	0.95–2.60
Contact with child services	14.8 (8)	**3.30**	1.62–6.75	**2.48**	1.17–5.24
Parental alcohol abuse	6.7 (3)	1.36	0.43–4.27	0.88	0.27–2.90
Parental mental illness	6.6 (5)	1.35	0.55–3.29	0.74	0.28–1.94

**Table A3:** Sensitivity analysis. Associations between family relations in adolescence and mortality from unnatural causes of death. Hazard ratios (HR) and 95% confidence intervals (95% CI) from Cox (proportional hazards) regressions. Results statistically significant at the 5%-level are reported in bold. (*n* = 2636).

Variable	% Deaths (n)	Unadjusted		Adjusted	
		HR	95% CI	HR	95% CI
Family relations					
Good (ref.)	1.7 (22)	1.00	-	1.00	-
Intermediate	3.5 (39)	**2.01**	1.19–3.39	**2.00**	1.19–3.39
Poor	4.2 (10)	**2.43**	1.15–5.13	**2.17**	1.01–4.66
Gender					
Males (ref.)	3.8 (52)	1.00	-	1.00	-
Females	1.5 (19)	**0.38**	0.23–0.65	**0.39**	0.23–0.66
Household social class					
Upper non-manual (ref.)	1.5 (9)	1.00	-	1.00	-
Interm./lower non-manual/					
entrepreneur/farmer	2.5 (26)	1.62	0.76–3.46	1.46	0.68–3.14
Manual worker	3.7 (36)	**2.43**	1.17–5.05	1.77	0.82–3.81
Household economic poverty	4.9 (16)	**2.10**	1.20–3.67	1.20	0.59–2.43
Contact with child services	14.8 (8)	**6.60**	3.16–13.77	**3.56**	1.61–7.86
Parental alcohol abuse	13.3 (6)	**5.58**	2.42–12.88	**3.79**	1.42–10.11
Parental mental illness	3.9 (3)	1.49	0.47–4.75	0.71	0.21–2.45

**Table A4:** Sensitivity analysis. Associations between family relations in adolescence and all-cause mortality in adulthood (26-65 years), controlling for psychiatric care in early adulthood (16-25 years). Hazard ratios (HR) and 95% confidence intervals (95% CI) from Cox (proportional hazards) regressions. Results statistically significant at the 5%-level are reported in bold. (*n* = 2619).

Variable	% Deaths (*n*)	Unadjusted		Adjusted	
		HR	95% CI	HR	95% CI
Family relations					
Good (ref.)	6.3 (80)	1.00	-	1.00	-
Intermediate	9.2 (103)	**1.47**	1.10–1.96	**1.40**	1.04–1.88
Poor	14.3 (34)	**2.34**	1.57–3.50	**1.87**	1.24–2.83
Gender					
Males (ref.)	10.3 (138)	1.00	-	1.00	-
Females	6.2 (79)	**0.59**	0.45–0.77	**0.56**	0.43–0.75
Household social class					
Upper non-manual (ref.)	5.6 (33)	1.00	-	1.00	-
Interm./lower non-manual/					
entrepreneur/farmer	8.2 (86)	1.48	0.99–2.21	1.39	0.93–2.08
Manual worker	10.1 (98)	**1.84**	1.24–2.73	1.43	0.94–2.16
Household economic poverty	14.3 (46)	**2.00**	1.44–2.77	1.36	0.91–2.02
Contact with child services	25.5 (13)	**3.71**	2.12–6.50	**2.41**	1.34–4.34
Parental alcohol abuse	22.2 (10)	**3.17**	1.68–5.98	**2.03**	1.02–4.05
Parental mental illness	14.5 (11)	**1.85**	1.01–3.40	0.81	0.42–1.59
Psychiatric care in adolescence	36.0 (28)	**5.65**	3.75–8.52	**4.73**	3.09–7.24
